# A model of the ternary complex of interleukin-10 with its soluble receptors

**DOI:** 10.1186/1472-6807-5-10

**Published:** 2005-06-28

**Authors:** Sergei Pletnev, Eugenia Magracheva, Alexander Wlodawer, Alexander Zdanov

**Affiliations:** 1 Macromolecular Crystallography Laboratory, Center for Cancer Research, National Cancer Institute at Frederick, Frederick, MD21702-1201, USA; 2 Basic Research Program, Science Application International Corporation-Frederick, National Cancer Institute at Frederick, Frederick, MD21702-1201, USA

## Abstract

**Background:**

Interleukin-10 (IL-10) is a cytokine whose main biological function is to suppress the immune response by induction of a signal(s) leading to inhibition of synthesis of a number of cytokines and their cellular receptors. Signal transduction is initiated upon formation of a ternary complex of IL-10 with two of its receptor chains, IL-10R1 and IL-10R2, expressed on the cell membrane. The affinity of IL-10R1 toward IL-10 is very high, which allowed determination of the crystal structure of IL-10 complexed with the extracellular/soluble domain of IL-10R1, while the affinity of IL-10R2 toward either IL-10 or IL-10/sIL-10R1 complex is quite low. This so far has prevented any attempts to obtain structural information about the ternary complex of IL-10 with its receptor chains.

**Results:**

Structures of the second soluble receptor chain of interleukin-10 (sIL-10R2) and the ternary complex of IL-10/sIL-10R1/sIL-10R2 have been generated by homology modeling, which allowed us to identify residues involved in ligand-receptor and receptor-receptor interactions.

**Conclusion:**

The previously experimentally determined structure of the intermediate/binary complex IL-10/sIL-10R1 is the same in the ternary complex. There are two binding sites for the second receptor chain on the surface of the IL-10/sIL-10R1 complex, involving both IL-10 and sIL-10R1. Most of the interactions are hydrophilic in nature, although each interface includes two internal hydrophobic clusters. The distance between C-termini of the receptor chains is 25 Å, which is common for known structures of ternary complexes of other cytokines. The structure is likely to represent the biologically active signaling complex of IL-10 with its receptor on the surface of the cell membrane.

## Background

IL-10 is a pleiotropic cytokine (reviewed in [1,2]) that suppresses immune system response by inhibiting synthesis of proinflammatory cytokines and their cellular receptors [3,4]. In addition to immunosuppressive functions, IL-10 also possesses immunostimulatory activities, including stimulation of growth and proliferation of thymocytes, cytokine-activated T-cells, mast cells, and B-cells [5-9].

Signal transduction is initiated when IL-10 binds to its cellular receptor, which consists of two chains, IL-10R1 and IL-10R2. IL-10 is an intercalated dimer made of two identical polypeptides, each 160 amino acids long [10-12], packed in two compact, six-helix bundle domains (reviewed in [2,13-15]). The domains are related by a twofold symmetry axis passing through the interdomain interface. Both receptor chains have about a 200 amino acids-long extracellular domain, a 20 amino acids-long transmembrane helix, and an intracellular/cytoplasmic domain, which contains 322 or 62 amino acids for IL-10R1 and IL-10R2, respectively. When the ternary complex IL-10/IL-10R1/IL-10R2 is formed, tyrosine kinases Tyk2 and Jak1 become activated and phosphorylate specific tyrosine residues on signal transducers and activators of transcription, which eventually leads to activation of the transcription of corresponding genes [16,17]. Kinetic binding data on soluble IL-10 receptor chains (sIL-10R) showed that sIL-10R1 exhibits nanomolar binding constants for IL-10, while sIL-10R2 does not display any significant affinity for IL-10, although binding of sIL-10R2 to a preformed binary IL-10/sIL-10R1 complex is noticeably better [18]. It has been commonly accepted that at first IL-10 binds to its high affinity receptor IL-10R1, forming a binding site for IL-10R2, and then the binding of the latter receptor finalizes the ternary signaling complex. It has been also shown for the IFN-(/receptor complex, which is likely to be similar to the IL-10 complex, that under physiological conditions the receptor chains are already preassembled together, even before the ligand binds. Thus, binding of the ligand is necessary only for opening the intracellular domain regions to accommodate intracellular components of the downstream signal transduction pathway [19]. It was shown for IL-19 and IL-20, which also belong to the IL-10 family, that their final ternary complexes with the soluble receptors are very much alike, no matter what was the order of binding components in solution [20]. Therefore, the structure of the ternary IL-10/sIL-10R1/sIL-10R2 complex can be predicted on the basis of the structures of the intermediate binary complex IL-10/sIL-10R1 and the structure of sIL-10R2. The former, stable complex was crystallized [21] and its structure was determined [22]; however, all attempts to obtain stable ternary complexes suitable for crystallization have so far failed.

The known structures of binary and ternary cytokine-receptor complexes show that although they differ from each other in details, their overall organization is somewhat similar [22-29]. What is even more important, both ligand/receptor and receptor/receptor binding sites are located in topologically similar areas on the surface of the interacting proteins and involve similar structural elements of the molecules (Table 1). For example, in the case of the ligands, only two major receptor binding sites were found, and both included topologically conserved N- and C-terminal helices that form a left-handed four-helix bundle [30], which is a signature element of all helical cytokines, as well as loop AB. The difference was that in some cases site I was used as a high-affinity receptor binding site, while in other cases site II was the one where the high-affinity receptor was found to bind (Table 1). The crystal structure of a binary complex of IL-10 with sIL-10R1 showed that the high-affinity receptor binding site is site I, and it is reasonable to assume that sIL-10R2 will bind IL-10 in site II, making receptor/receptor contacts similar to the already published structures of ternary complexes.

We generated a structural model of the ternary complex of IL-10 with its two soluble receptors, sIL-10R1 and sIL-10R2. The model has been built on the assumption that the structure of the binary IL-10/sIL-10R1 complex must be preserved upon the binding of the second receptor chain, the complex must possess twofold symmetry, and that the structure of the complex of one domain of IL-10 with sIL-10R1 and sIL-10R2 should be somewhat similar to the already known structures of ternary complexes having two different receptor chains. To fulfill the latter requirement, we took the structure of IL-6/IL-6Rα /gp130 [29] as an example.

## Results

### Structure of sIL-10R2

**Figure 1:**
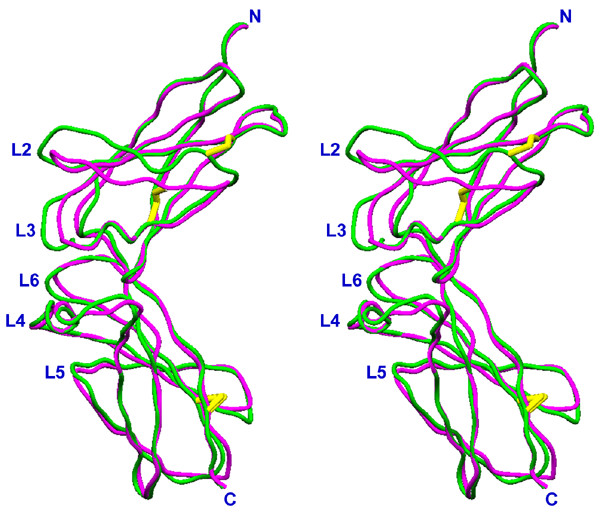
Stereo diagram of the superposition of sIL-10R1 and sIL-10R2. sIL-10R1 is green, sIL-10R2 is magenta, the disulfide bonds are shown in yellow.

**Figure 2:**
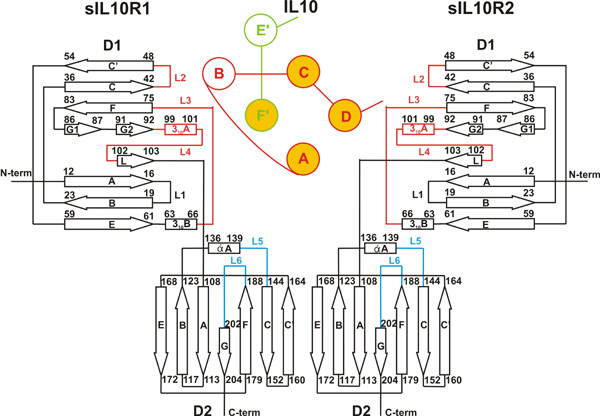
Topology diagram of a single ligand domain associated with high- and low-affinity receptors. β-strands of the receptor molecules are shown as arrows and the helices are shown as rectangles. Binding loops of the receptor D1 domain are red and the loops of the D2 domain are blue. α-helices of single ligand domain are shown as circles. Helices A, B, C, and D (red) belong to one polypeptide chain of the ligand and helices E' and F' (green) belong to another polypeptide chain of the ligand. Highlighted helices (A, C, D, F') constitute the four-helix bundle that is involved in receptor binding.

The structures of sIL-10R2 and sIL-10R1 appear to be very similar (Fig. 1). They are both L-shaped molecules consisting of two elongated N- and C-terminal domains (D1 and D2), oriented at about 90° with respect to each other, each having FBN-III-like topology [31-33]. Each domain consists of seven β-strands organized in two antiparallel β-sheets – A, B, E and C, C', F, and G – that pack one against another in the shape of a sandwich. Loops L2-L4 of D1 and L5-L6 of D2 (Fig. 2) are involved in ligand binding. Domains D1 and D2 of sIL-10R2 comprise 96 and 94 amino acid residues, respectively (residues 1–103 and 109–206), and are connected to each other by a 5 amino acid-long linker (residues 104–108).

**Figure 3:**
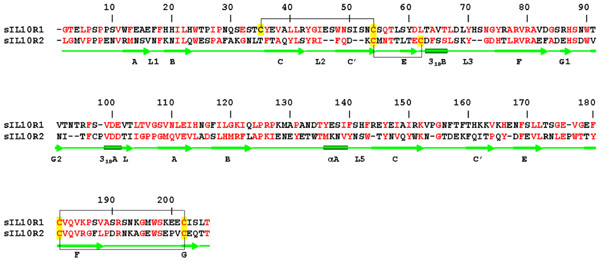
Structure-based sequence alignment of soluble receptors sIL-10R1 and sIL-10R2. Aligned molecules have 20.4% of identical residues and 53.1% of homologous residues (red). Secondary structure elements are shown in green. Cysteines involved in disulfide bonds are highlighted in yellow, disulfide bonds are shown by black lines.

Deletions in the amino acid sequences of loops L2, L3 and β-strand C' in domain D1 of sIL-10R2 (Fig. 3) make its structure more compact than sIL-10R1. As a result, Tyr43, which is a conserved residue in class II receptors [34], is located a little higher than its counterpart in sIL-10R1. Although in sIL-10R1 Tyr43 plays a very important role in the interaction with the ligand, in sIL-10R2 it is located at the very edge of the receptor/ligand interface. Cysteines 54 and 62 of sIL-10R2 form a disulfide bond that is conserved in class II receptors [32], linking β-strand C' with the loop between the strand E and helix B. An equivalent disulfide bridge in sIL-10R1 is formed between cysteines 54 and 35 [12,22], linking together β-strands C' and C (Fig. 1).

Loop L3 of sIL-10R2 is two amino acids shorter in sIL-10R1 and does not protrude out of the D1 domain as much as in sIL-10R1. This alters the shape of the N-terminal part of the interdomain region and makes it complementary to the surface of the ligand-binding site II. Tyr70 is shifted ~2.5 Å inside of the D1 domain to compensate for the shortage of loop L3. In both receptors, the side chain of Tyr70 participates in the formation of a hydrophobic core of the N-terminal domain. Deletions in strand C' and loop L3 make the N-terminal domain of sIL-10R2 about 3 Å shorter than in sIL-10R1.

β-strands F (residues 77–81) of both receptors contain a conserved sequence (A/L)RVRA. Together with the sequences HSDWV in sIL-10R2 and HSNWT in sIL-10R1 (residues 87–91), this motif makes an extended π-cation system [35,36], usually found in the C-terminal domains of the type I family of cytokine receptors [37]. In sIL-10R1, a stacking structure is formed by His87, Arg80, Trp90, Arg78, Trp48, and Leu41; and is about 19 Å in length, which corresponds to roughly half of the domain length (~ 36 Å). It connects β-strands C, C', F, and G, and stabilizes the structure of the domain. In sIL-10R2, β-strand C' is located away from the β-strand F, which makes it impossible for the side chains of Phe48 and Leu41 to participate in the formation of a π-cation system, consisting in this case of only four residues – His87, Arg80, Trp90 and Arg78 – that keep together β-strands G and F. Although the π-cation system of sIL-10R2 is only ~11 Å long, which is about one-third of the total length of the domain, it still may be important for the stability of the structure of the domain. It is also likely that the π-cation system may play a dual role during the lifetime of a cytokine receptor. Mutagenesis studies of the erythropoietin receptor revealed the importance of the π-cation system for the transport of the receptor from endoplasmic reticulum to the Golgi apparatus [38,39].

In comparison to D2 of sIL-10R1, D2 of sIL-10R2 has three single amino acid deletions: Arg144, Pro154, and Glu167, as well as a single insertion of Pro176A (Fig. 3). The first deletion makes the connection between loop L5 and β-strand C shorter and moves the residues of loop L5 about 0.5 Å closer to the main body of the domain. Similarly, deletions of Pro154 and Glu167 make the loops connecting strands C and C' and strands C' and E slightly shorter (Fig. 1). β-strands F and G are held together by a disulfide bridge between Cys181 and Cys202, which is a common feature for class II cytokine receptors. Insertion of Pro176A in sIL-10R2 extends the loop between β-strands E and F by about 3 Å, bringing this part of the D2 domain closer to the cell membrane.

### Hydrophobic patches on the surface and intermolecular hydrophobic clusters of the ternary IL-10/sIL-10R1/sIL-10R2 complex

Both the ligand and receptor chains have several hydrophobic patches located on the surface of the molecules (Table 2). Although the surface of IL-10 is mostly hydrophilic, with 86% of hydrophobic residues involved in the formation of the internal hydrophobic core [13], it also has three solvent-exposed hydrophobic patches (Table 2). Patch 1 is involved in the binding of sIL-10R1; patch 2 interacts with both sIL-10R1 and sIL-10R2, while patch 3 is located on the surface of helices A and D of IL-10 and participates in interactions with sIL-10R2.

sIL-10R1 contains six hydrophobic patches, three in the N-terminal domain and three in the C-terminal domain (Table 2). Patches 1 and 2 are similar in size and located on the opposite sides of the domain. Patch 1 is formed by five residues coming from β-strands C' and E and helix B, as well as three residues of the C-terminal domain flexible loop connecting β-strand B and helix A. This is the largest hydrophobic region in the first domain sIL-10R1, covering the surface of 475 Å^2^. Patch 2 is formed by seven residues coming from β-strands A, B, and E, and covers the surface of 448 Å^2^. Thr25 is at the center of the patch and although it is not a hydrophobic residue, its side chain is oriented in such a way that Oγ 1 points inside the protein molecule and forms a hydrogen bond with the main chain nitrogen of Ser10, whereas Cβ and Cγ 2 atoms are exposed to solvent. Patch 3 consists of three residues of loop L2 and covers the surface of 207 Å^2 ^in the interdomain region of sIL-10R1. Patch 4 consists of eight residues covering the surface of 546 Å^2 ^and is located close to the C-terminus, near the area which is likely to be in contact with the cellular membrane *in vivo *(Table 2). Patch 5 includes six residues coming from β-turn CC' and the first half of β-strand C'. It covers the surface of 409 Å^2 ^and has an elongated shape. Patch 6 is formed by three residues of loop L5 and covers 253 Å^2 ^of the sIL-10R1 interdomain region.

The pattern of hydrophobic patches of sIL-10R2 is similar, although not identical, to that of sIL-10R1. sIL-10R2 has seven patches, three in the N-terminal domain and four in the C-terminal domain. Patch 1 lies at the top of the sIL-10R2 N-terminal domain and is composed of 10 residues covering the surface of 635 Å^2^. This is the largest hydrophobic solvent-exposed area on the surface of sIL-10R2. Patches 2 and 3 (Table 2) cover 284 Å^2 ^and 154 Å^2^, respectively, and are located in the proximity of the ligand/receptor interface. Patch 4 is formed by seven residues of β-strands C' and E, filling the gap between the strands and shielding the internal hydrophobic core of the molecule from the solvent. The area of the patch is 572 Å^2^. Patch 5 is formed by four residues coming from the β-turn connecting strands A and B, and is 274 Å^2 ^in size. Patches 4 and 5 are located at the very bottom of the C-terminal domain and are likely to be in contact with the cellular membrane. Patch 6 covers 203 Å^2 ^and is located in the central part of the receptor-receptor interface. Patch 7 consists of only two residues that belong to loops L5 and L6. It is 178 Å^2 ^in size and is a part of the IL-10/sIL-10R2 interface.

**Figure 4:**
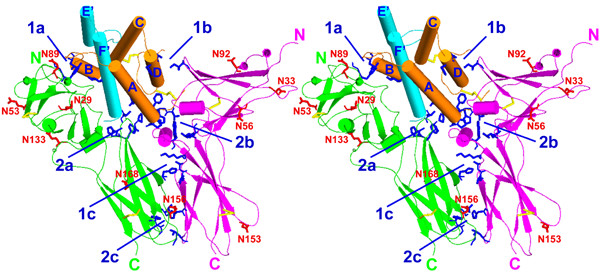
Stereo diagram of intermolecular hydrophobic clusters of the ternary IL-10/sIL-10R1/sIL-10R2 complex. Polypeptide chains of IL-10 are shown in orange and cyan, sIL-10R1 is green, and sIL-10R2 is magenta. Side chains of the residues that constitute hydrophobic clusters are shown in blue. Clusters 1a and 2a are the "top" and the "bottom" hydrophobic regions of IL-10/sIL-10R1 interface, clusters 1b and 2b are the "top" and the "bottom" hydrophobic regions of IL-10/sIL-10R2 interface and clusters 1c and 2c are the "top" and the "bottom" hydrophobic regions of sIL-10R1/sIL-10R2 interface. Disulfide bonds are shown in yellow. The potential glycosylation sites are shown in red.

Upon formation of the ternary complex, hydrophobic patches from different molecules compensate each other, creating intermolecular hydrophobic clusters inside of ligand-receptor and receptor-receptor interfaces (Table 2, Fig. 4). Thus, one IL-10 domain complexed with two receptor chains has two such clusters (1a and 2a, Fig. 4) in the IL-10/sIL-10R1 interface, two clusters (1b and 2b) in the IL-10/sIL-10R2 interface, and two clusters (1c and 2c) in the sIL-10R1/sIL-10R2 interface. Intermolecular hydrophobic clusters found in the ternary IL-10/sIL-10R1/sIL-10R2 complex are important elements that hold molecules together after they have attracted each other by long-range ionic interactions. It is worthwhile to note that patch 1 and 2 of sIL-10R1 and patches 1, 3 and 4 of sIL-10R2 remain exposed to solvent on the surface of the ternary complex.

### The structure of the IL-10/sIL-10R1/sIL-10R2 complex

**Figure 5:**
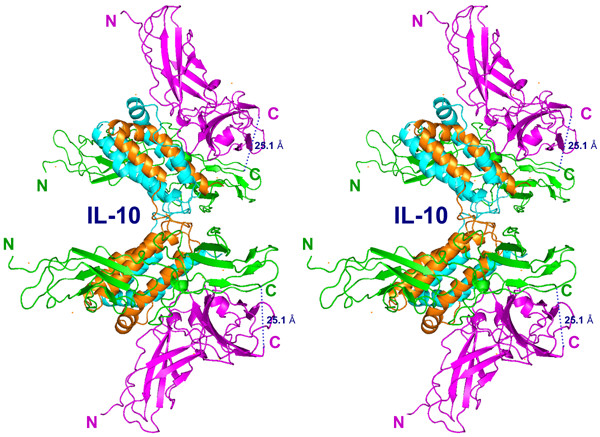
Stereo diagram of the ternary complex of human IL-10/sIL-10R1/sIL-10R2. Polypeptide chains of IL-10 are shown in orange and cyan, sIL-10R1 is green, sIL-10R2 is magenta. The hypothetical cell membrane is perpendicular to the plane of the figure on the right and the twofold symmetry axis (not shown) is horizontal.

**Figure 6:**
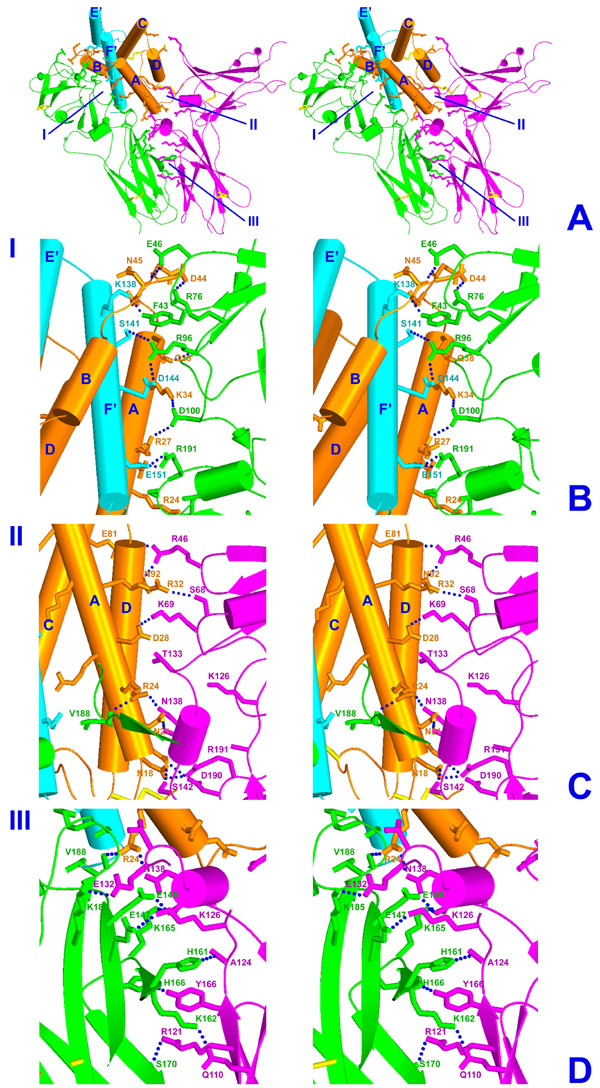
Stereo diagram of each interface within a single IL-10/sIL-10R1/sIL-10R2 signaling unit (panel A). Polypeptide chains of IL-10 are shown in orange and cyan. sIL-10R1 is green and sIL-10R2 is magenta. Panels B, C and D represent close-up view of each interface, including contact residues. (B)- an interface between IL-10 and sIL-10R1, (C)- an interface between IL-10 and sIL-10R2, (D)- an interface between sIL-10R1 and sIL-10R2. Intermolecular hydrogen bonds calculated with in 3.2 Å distance cutoff are shown as blue dotted lines.

The ternary complex of IL-10/sIL-10R1/sIL-10R2 is a twofold symmetrical molecule in which each of the domains of IL-10 is bound with two receptor chains (Fig. 5). The receptor chains are bound to adjacent sides on the surface of a single IL-10 domain (Table 1). The sIL-10R1 binding site (site I) is formed by helix A, loop AB, and helix F', while the sIL-10R2 binding site (site II) is made by helices A and D (Fig. 6). sIL-10R1 and sIL-10R2, bound to the same IL-10 domain, interact with each other by their D2 domains, forming a receptor-receptor binding site (site III, Fig. 6). The distance between C-termini of sIL-10R1 and sIL-10R2 is 25.1 Å, which lies within the 25–32 Å range found in the structures of other ternary complexes [23-25,29]. The structure of the binary/intermediate complex of IL-10/sIL-10R1 [22] is the same in the ternary complex. We found only minor movements of some side chains involved in the interactions with sIL-10R2 in sites II and III. Both sIL-10R1 and sIL-10R2 receptor chains interact with ligand loops L2-L6. Site III involves interactions between residues coming from β-strands C, C', E, F and loop L6 of sIL-10R1, and residues from β-strands A, B, E and loop L5 of sIL-10R2 (Figs. 2, 6).

### Site I interface (IL-10/sIL-10R1)

The interaction of IL-10 with sIL-10R1 in the ternary complex is the same as that found in the crystal structure of the intermediate/binary complex [22]. Briefly, the interface is formed by the residues of the second half (middle and C-terminal) of helix A, loop AB, and helix F' of IL-10, as well as loops L2-L6 of sIL-10R1. Twenty-seven residues of the ligand interact with 23 residues of the receptor chain, creating an extensive network of hydrogen bonds (70%) (Table 3, Figs. 6A, 6B) and hydrophobic interactions (30%) (Table 2). The total change in the accessible surface for both IL-10 and sIL-10R1, calculated using a spherical probe of the radius 1.40 Å, is about 2116 Å^2^. Thus, the contact area between IL-10 and sIL-10R1 is about 1058 Å^2^. The most important residues involved in receptor-ligand binding are Pro20, Arg24, Arg27, Gln38, Glu42, Lys138, Ser141, Asp144, Glu151, and Ile158 of IL-10 and Tyr43, Arg76, Arg96, Phe143, Ser190 and Arg191 of the sIL-10R1 [22]. The IL-10/sIL-10R1 interface includes two hydrophobic clusters located at the top and at the bottom of the interface (Table 2, Fig. 4).

### Site II interface (IL-10/sIL-10R2)

The interface of site II is formed by helix A, loop CD, and helix D of IL-10, as well as by loops L2, L3, L6, and α-helix A of the sIL-10R2 (Table 2, Figs. 6A, 6C). Helix A interacts with loops L3, L6, and helix A, whereas helix D interacts with loop L2 of sIL-10R2. Site II is smaller than site I; its total surface is 568 Å^2^, which is only 54% of the surface of site I. IL-10 and sIL-10R2 each donate 13 residues to the interface. Interacting residues are mostly polar (~80%) and link the ligand to the receptor via hydrogen bonds (Table 3) and hydrophobic interactions (Table 2). The most important interface residues are Pro16, Asn18, Asn21, Arg24, Asp28, Arg32, Glu81, Ala89, His90, and Asn92 of IL-10 and Arg46, Ile47, Ser68, Lys69, Thr133, Asn138, Ser142, Trp143, and Arg191 of sIL-10R2. Most interactions occur between IL-10 helix A and loops L3 and L6 of sIL-10R2, with only a few contacts between IL-10 helix D and loop L2 of the sIL-10R2. Arg24 of IL-10 occupies a unique position in the complex. Its side chain projects down towards the cell membrane along the receptor-receptor interface and forms hydrogen bonds with the carbonyl oxygen of Val188 of sIL-10R1 and the side-chain atom Oδ 1 of Asn138 of sIL-10R2, linking both receptor chains together. The IL-10/sIL-10R2 interface also includes a hydrophobic cluster located at the bottom part of the interface, formed by Pro16 of the ligand and Trp143 of the receptor. The upper part of the IL-10/sIL-10R2 interface also includes a hydrophobic cluster formed by Ala89 and His90 of IL-10, and I47 of sIL-10R2. Unlike in the cluster 2c (Fig. 4), the hydrophobic area 1c is weaker but still is an important element of the IL-10/sIL-10R2 interface. A smaller interface area and a reduced amount of interacting residues reflect the low affinity of the sIL-10R2 towards its binding partners.

### Site III interface (sIL-10R1/sIL-10R2)

The interface between sIL-10R1 and sIL-10R2 is formed by residues that belong to β-strands C, C', E, and F of sIL-10R1 and by residues of β-strands A, B, E, and loop L5 of sIL-10R2 (Tables 2, 3; Fig. 6D). The surfaces of C-terminal domains of the receptors are complementary. Within 3.8 Å distance cutoff, receptors 1 and 2 donate 15 and 13 residues, respectively, to form a contact area that is composed of 65% hydrophilic and 35% hydrophobic residues and is about 803Å^2 ^in size (Tables 2, 3). The most important residues are Gly116, Phe117, Glu145, Glu147, His161, Lys162, Lys165, His166, Ser170, Leu172, Gly175, and Lys185 of sIL-10R1, and Gln110, Leu114, His119, Arg121, Leu123, Ala124, Lys126, Glu132, Asn138, Asn141, and Tyr166 of sIL-10R2. The contact area between two receptors can be divided into three parts. The upper part of site III is hydrophilic and contains a wide network of hydrogen bonds. Almost all residues that are located in this area are hydrophilic. The middle part of the interface has a roughly equal number of charged and uncharged residues, while the lower part of the receptor/receptor interface is almost solely hydrophobic, with an intermolecular hydrophobic cluster at the bottom of C-terminal domains of the receptors (Figs. 4, 6).

### Glycosylation sites

The receptors sIL-10R1 and sIL-10R2 contain six and four potential N-linked glycosylation sites, respectively (Fig. 4). In the complex, all sites are fully exposed to the solvent and are not part of receptor-ligand or receptor-receptor interfaces. Moreover, each particular potential carbohydrate-binding site is located at a sufficient distance from the interfaces, which eliminates the possibility of a sugar chain clashing with a neighboring protein molecule. In sIL-10R1, Asn29 flanks the top end of the receptor N-terminal domain, whereas Asn53 and Asn89 are in the central part of the domain. They are located on loop BC, β-strand C', and in the region between β-strands G1 and G2, respectively. The N-terminal domain of sIL-10R2 also has three potential glycosylation sites: Asn33, Asn56, and Asn92. Asn33 is located close to the N-terminal end of the domain, whereas Asn56 and Asn92 are found in the central part of the domain, on the opposite sides of a β-sandwich.

The C-terminal domain of sIL-10R1 contains three potential glycosylation sites at Asn133, Asn156, and Asn168, located near the α-helix A, on β-turn CC', and at the beginning of β-strand E (Figs. 2, 4), respectively. The site at Asn133 is located near the N-terminal end of the domain, close to the interdomain region of sIL-10R1. Asn156 is at the bottom of the C-terminal domain, while the oligosaccharide attached to Asn168 is in the central part of domain D2. In the ternary complex, this residue is likely to be in contact with the cellular membrane. The C-terminal domain of sIL-10R2 has only one potential carbohydrate site, Asn153, which is close to the C-terminus of the receptor. This residue is a part of β-turn CC' and is very close to position Asn156 of sIL-10R1 upon superposition of the receptor chains.

## Discussion

It is clear that the quality of any theoretical model should be assessed based on its agreement with experimental data such as, for example, mutagenesis. Unfortunately, we were unable to find any such data for the IL-10/IL-10R1/IL-10R2 complex, either in literature or as personal communications. Therefore, to evaluate the correctness of the model, we can only rely on the commonly accepted criteria: the model is in the global minimum of energy; general similarity to other ternary complexes of cytokines; usage of similar, well defined receptor binding sites on the surface of the ligand (Table 1); and correlation between the available kinetic binding data and intermolecular binding surfaces and contacts. As we have already mentioned, a general similarity of the ternary IL-10 complex to known structures of other ternary complexes and involvement of the similar ligand binding sites have been postulated from the beginning; thus, these conditions have been necessarily satisfied. The model of the ternary complex does correspond to the minimum of the energy, and the pattern of the interactions of sIL-10R2 with the binary IL-10/sIL-10R1 complex agrees well with its low affinity nature. Recent study of binding of IL-10 to IL-10R2 by peptide scans [40] showed that, although IL-10R2 did not form a binary complex with IL-10, it recognized regions of helix A and loop AB of IL-10 (amino acid residues 19–43) when they were presented as 15-mer peptides. In our model, the IL-10/IL-10R2 interface includes residues 18–32 of IL-10 (Table 3). Among the residues involved in creating the IL-10R1/IL-10R2 interface, seven residues of IL-10R1 have the same residue type as residues 33–43 of IL-10 (Table 2, Table 3). This may explain the necessity of formation of IL-10/IL-10R1 binary complex before IL-10R2 can join and complete the ternary signaling complex.

IL-10 binds to the receptor chains via two sites located on the adjacent sides of its four-helix bundle [30]. Site I is formed by helices A and F', whereas site II is formed by helices A and D (Table 1). Both receptor chains interact with IL-10 through their cytokine recognition motifs, which consist of the loops and β-turns of the receptor interdomain region connecting consecutive antiparallel β-strands [22-29]. Typically, the low-affinity receptor cannot bind to a ligand by itself. Its association occurs via a cooperative binding site that is formed by both the ligand and the high-affinity receptor. With the exception of the prolactin ternary complex, C-terminal domains of the receptors contact each other in the vicinity of the cellular membrane (Table 1). Such interaction brings the intracellular parts of the receptors together, resulting in activation of signal transduction.

Receptor chains of the ternary IL-10 complex interact with each other via β-strands C, C', E, and F of sIL-10R1 and β-strands E, B, A, and loop L5 of sIL-10R2, forming an interface between the sides of their C-terminal domains Tables (2, 3; Figs. 4, 5, 6). The distance between the Cα atoms of the C-termini of IL-10R1 and IL-10R2 is 25.1 Å. In the known structures of other ternary complexes, the distances between the Cα atoms of the last residues of the extracellular parts of the receptor chains vary between 25 and 32 Å.

The surfaces of IL-10 and of both the sIL-10R1 and sIL-10R2 receptors contain several hydrophobic patches (Table 2). In the ternary complex, most of these areas face each other to form intermolecular hydrophobic clusters. Each interface contains two such areas that flank opposite sides of contact regions (Fig. 4). The bottom clusters of IL-10/sIL-10R1 and IL-10/sIL-10R2 touch each other at the point where the ligand and both receptors join, creating a larger hydrophobic area formed by all three molecules. Such a cluster is not observed in other structures of ternary complexes and is, therefore, unique for the ternary IL-10 complex.

Both receptors have several potential glycosylation sites, which are located on protein surfaces in places where they do not conflict with the three-dimensional organization of the IL-10/sIL-10R1/sIL-10R2 complex (Fig. 4). The role of carbohydrates in forming and maintaining cytokine/receptor complexes is not well understood. It was shown that the granulocyte-macrophage colony-stimulating factor (GM-CSF) receptor α subunit requires N-glycosylation for binding and signaling [41]. Tunicamycin treatment inhibited GM-CSF binding in a dose-dependent manner, with a maximum of 85% inhibition at 3 μg/ml. Treatment of human leukemia HL-60 cells with tunicamycin completely blocked GM-CSF-induced tyrosine phosphorylation, which suggests that N-glycosylation of the receptor is necessary for intracellular signaling [41]. However, the non-glycosylated mutant of sIL-10R1 is capable of forming a stable binary complex with IL-10 [22]. GM-CSF is a glycoprotein in which glycosylation is not required for its biological activity. GM-CSF that is expressed in *E. coli *and thus, not glycosylated, retains full activity [42]. By contrast, the IL-10R2 binding region on IL-22 contains an N-linked carbohydrate that is likely important for binding [43]. Similarly, N-linked glycosylation of viral IL-6 is shown to increase gp130 binding and biological activity [44]. Taking into account that no carbohydrates are involved in the intermolecular interactions in the ternary IL-10/sIL-10R1/sIL-10R2 complex, as well as the fact that deglycosylated sIL-10R1 can form a quite stable binary complex with the IL-10, we may conclude that N-linked oligosaccharides are not essential for the formation of the biologically active ternary IL-10/IL-10R1/IL-10R2 complex. It is clear that more structural data are required to address the question of why some cytokines and their receptors depend upon the presence of carbohydrates, whereas others do not.

It was shown that a series of anti-human IL-10 antibodies can efficiently neutralize this cytokine [45]. Based on the recognition epitope, antibodies can be divided into three groups, A, B and C. The epitopes were identified using IL-10-derived, overlapping peptide scans prepared by spot synthesis. Antibodies of group A inhibit biological activity of IL-10 in an approximately equimolar ratio, at concentrations as low as 10 pM [45]. It has been shown that monoclonal antibody CB/RS/2 recognizes two binding regions of a discontinuous epitope on the surface of the molecule that comprises the N-terminal half of helix A and helix D [46]. Thus, this antibody binds to site II of the ligand and prevents IL-10 from association with its low-affinity receptor. An overlapping peptide scan technique has also been used for mapping IL-10 residues that bind to sIL-10R1, as well as mapping the residues of sIL-10R1 that interact with the ligand [47]. It was shown that residues of helices A and C of the ligand interact with the residues of loops L3-L6 of the high-affinity receptor. The structure of the IL-10/sIL-10R1 complex confirmed those results [22]. However, no mapping data exist for the residues that form the IL-10/sIL-10R2 interface. Such data would be very valuable to guide the design of biochemical experiments.

In the absence of sIL-10R1, IL-10 cannot bind to sIL-10R2, and the two receptor chains cannot interact with each other without a ligand. Therefore, assembly of the ternary complex consists of two steps. In the first step, IL-10 binds to sIL-10R1, forming a binary complex; subsequently, sIL-10R2 binds to the preformed IL-10/sIL-10R1 complex, completing the ternary complex. Such differences in binding abilities could be linked to the physical characteristics of the corresponding interfaces. The area of site I (1058 Å^2^) is about 25–45% larger than the areas of site II (568 Å^2^) and site III (803 Å^2^). The number of residues involved in interface formation is also larger for the IL-10/sIL-10R1 contact region. Site I is formed by a total of 50 residues, whereas sites II and III are composed of 26 and 28 residues, respectively. However, when combined together, sites II and III have an area and number of charged and hydrophobic interactions comparable to those of site I, which may explain the ability of the low-affinity receptor to bind to a preformed ligand-high-affinity receptor complex. This is also true if we compare the areas of binding sites I, II and III in other ternary complexes of cytokines such as GH, PL, EPO, and IL-6; there, the area of site I is usually larger than each of the areas of site II or III, whereas combined area of sites II and III are roughly equal to or larger than the area of site I [23,25,24,29].

## Methods

The model of sIL-10R2, the low-affinity receptor, was generated using the published crystal structure of sIL-10R1 as a template (pdb code 1J7V ([22]). The original amino acid sequence of sIL-10R1 was mutated to that of sIL-10R2 in accordance with the alignment of their sequences shown in Figure 3 (20.4% identity and 53.1% similarity) [34,48]. Model building was followed by energy minimization, which included 500 cycles of positional refinement to optimize stereochemistry of newly built parts of the structure, followed by simulated annealing slow cool protocol and another 500 cycles of positional refinement. To preserve the overall fold, a 2.8 kcal/mole restraint has been imposed on all Cα atoms of the model. All other main and side-chain atoms were allowed to move freely within the appropriate stereochemical parameters.

A model of the ternary complex of one domain of IL-10 with sIL-10R1 and sIL-10R2 was generated on the assumption that the structure of the intermediate binary complex IL-10/sIL-10R1 does not change much upon binding of the second receptor chain, sIL-10R2, and that mutual arrangement of ligand and receptor chains is similar to what was found in the crystal structure of IL-6/IL-6-Rα /gp130 [29]. At the first step, the Cα atoms of the structure of one domain of IL-10 complexed with sIL-10R1 were superimposed onto Cα atoms of IL-6/sIL-6Rα, and then the structure of sIL-10R2 was superimposed onto gp130. Subsequently, the best fit of charge complementarity of sIL-10R2 to the intermediate binary complex was achieved upon superposition of the C-terminal domain of sIL-10R2 with the C-terminal domain of gp130, followed by rotation of sIL-10R2 as a whole around the axis perpendicular to the cell surface and passing through the center of mass of the IL-10 domain, followed again by translation along the surface of helices A and D. The second half of the hexameric IL-10/sIL-10R1/sIL-10R2 complex was generated by applying twofold symmetry (Fig. 5). Model building was followed by the energy minimization procedure under conditions described above until the drop in total energy between consecutive refinement steps was less than 1%. Root mean square deviations in positions of Cα atoms for IL-10, sIL-10R1 and sIL-10R2 in the ternary complex before and after energy minimization were 0.38 Å, 0.40 Å and 0.45 Å, respectively.

Sequence alignment was performed with program CLUSTALW 1.74 [48], model building with program CHAIN [49], and energy minimization with XPLOR 3.1 [50]; all superpositions were performed with program LSQKAB from CCP4 [51]. Figures 1, 4, and 6 were generated with program SETOR [52], while Figure 5 was generated with the program pyMOL (DeLano W.L. The PyMOL Molecular Graphics System 2002, DeLano Scientific, San Carlos, CA, USA).

## Authors' contributions

SP and EM performed amino acid sequence alignment, generated the model and participated in writing the manuscript. AW participated in discussions and writing the manuscript. AZ conceived the study and participated in all stages of the work. All authors read and approved the final manuscript.

## Figures and Tables

**Table 1:** Positions of the cytokine-receptor binding sites in known structures of ternary and binary complexes.

**Cytokine-receptor complexes (PDB code/reference)**	**Site I (Ligand/Receptor)**		**Site II (Ligand/Receptor)**		**Site III (Recept/Recept)**
	SSE* Ligand	SSE* Receptor	SSE* Ligand	SSE* Receptor	**Contacting Domains**

**Ternary complexes**					
hGH/GHbp/GHbp (3HHR [23])	A, loop AB, D	L1, L3-L6	A, C	L1, L3-L6	D2-D2
hEPO/sEPOR/sEPOR (1EER [24])	A, loop AB, D	L1-L6	A, C	L1-L3, L5, L6	no interaction
oPL/srPRLR/srPRLR (1F6F [25])	A, loop AB, D	L1-L6	A, C	L1-L4	D2-D2
hIL6/sIL6Rα /gp130 (D1-D3) (1P9M [29])	A, D	L2-L6	A, C	L1-L3, L5	D2-D3
**Binary complexes**					
hINF-γ /sINF-γ Rα ** (1FG9 [53])	A, loop AB, F'	L2, L3, L5, L6			
hLIF/gp130 (D2-D3) (1PVH [54])			A, C	L2, L3, L5	
G-CSF/G-CSFR (BN-BC) (1CD9 [27])			A, C	L2-L6	
hIL4/IL4-R1 (1IAR [26])			A, C	L1-L6	
hIL-10/sIL-10R1** (1J7V [22])	A, loop AB, F'	L2-L6			
cmvIL-10/sIL-10R1** (1LQS [55])	A, loop AB, F'	L2-L6			
vIL-6/gp130 (1I1R [56])			A, C	L2, L3, L5	
**Ternary hIL-10 complex**					
hIL10/sIL10R1/sIL10R2**,***	A, loop AB, F'	L2-L6	A, D	L2, L3, L5, L6	D2-D2

(*)- SSE: Secondary structure elements.

(**)- In INF-γ and IL-10 structures helix F' is a topological equivalent of helix D of other helical cytokines.

(***)- Interface areas were calculated with program Surface (CCP4) using spherical probe of the radius 1.40 Å^2^.

**Table 2:** Hydrophobic patches of IL-10, sIL-10R1 and sIL-10R2

**IL-10**		**Interaction site**
**Patch 1 ** Residue Access. (%)	**Surface area 189 Å**^2 ^ L46 L53 I145 A152 50 25 34 44	**IL-10/sIL-10R1 interface,** with patch 3 of sIL-10R1
**Patch 2 ** Residue Access. (%)	**Surface area 294 Å**^2 ^ H14 P16 P20 I158 73 66 71 25	**IL-10/sIL10R1 interface, ** with patch 6 of sIL-10R1 **IL-10/sIL10R2 interface, ** with Patch 7 of sIL-10R2
**Patch 3 ** Residue Access. (%)	**Surface area 183 Å**^2 ^ F36 A89 H90 33 86 45	**IL-10/sIL-10R2 interface ** with Patch 2 (sIL-10R2)
**IL-10R1 Domain D1**		
**Patch 1 ** Residue Access. (%)	**Surface area 475 Å**^2 ^ I51 Y60 A64 V65 L67 18 39 90 34 46 M129 P131 A132 60 38 100	**No interaction**
**Patch 2 ** Residue Access. (%)	**Surface area 448 Å**^2 ^ P6 P9 W12 T25 P26 P28 L58 45 45 45 39 56 65 56	**No interaction**
**Patch 3 ** Residue Access. (%)	**Surface area 207 Å**^2 ^ Y43 G44 I45 44 66 83	**IL-10/sIL10R1 interface, ** with patch 1 of IL-10
**Domain D2**		
**Patch 4 ** Residue Access. (%)	**Surface area 546 Å**^2 ^ I113 H114 G116 F117 60 56 47 58 L119 L172 G175 V177 41 84 61 69	**sIL-10R1/sIL10R2 interface, ** with patch 5 of sIL-10R2
**Patch 5 ** Residue Access. (%)	**Surface area 409 Å^2^** V153 P154 G155 F157 F159 H161 45 56 55 73 43 56	**sIL-10R1/sIL10R2 interface, ** with patch 6 of sIL-10R2
**Patch 6 ** Residue Access. (%)	**Surface area 253 Å^2^** H142 F143 A189 77 62 35	**IL-10/sIL10R1 interface, ** with patch 2 of IL-10
**IL-10R2 Domain D1**		
**Patch 1 ** Residue Access. (%)	**Surface area 635 Å^2^** L2 G3 M4 P6 P7 A28 77 78 45 56 40 91 F29 G32 F83 A84 97 93 34 85	**No interaction**
**Patch 2 ** Residue Access. (%)	**Surface area 284 Å^2^** L41 I47 F48 29 88 62	**IL-10/sIL10R2 interface, ** with patch 3 of IL-10
**Patch 3 ** Residue Access. (%)	**Surface area 154 Å^2^** C98 P98A V99 52 48 42	**No interaction**
**Domain D2**		
**Patch 4 ** Residue Access. (%)	**Surface area 572 Å^2^** F160 I162 F169 L172 73 26 12 71 L175 P176A W177 49 73 57	**No interaction**
**Patch 5 ** Residue Access. (%)	**Surface area 274 Å^2^** V113 L114 A115 H119 47 62 100 40	**sIL-10R1/sIL10R2 interface, ** with patch 4 of sIL-10R1
**Patch 6 ** Residue Access. (%)	**Surface area 203 Å^2^** P107 L123 A124 69 65 54	**sIL-10R1/sIL10R2 interface, ** with patch 5 of sIL-10R1
**Patch 7 ** Residue Access. (%)	**Surface area 178 Å^2^** W143 P189 57 48	**IL-10/sIL10R2 interface, ** with patch 2 of IL-10

**Table 3:** Intermolecular hydrogen bonds of the IL10/sIL10R1/sIL10R2 complex

IL-10-IL-10R1 interface				
IL-10	SSE	IL-10R1	SSE	Distance **Å**
R24L NE	A	R191R O	L6	3.0
R24L NH1	A	V188R O	L6	2.9
R24L NH1	A	R191R O	L6	3.0
R27L NE	A	S190R O	L6	2.9
R27L NH1	A	D100R OD2	L4	3.2
K34L NZ	A	D100R OD1	L4	2.9
Q38L OE1	A	R96R N	L4	3.0
D41L O	loop AB	R76R NH1	L3	2.9
Q42L O	loop AB	R76R NH2	L3	3.0
D44L OD1	loop AB	R76R NH2	L3	2.8
D44L O	loop AB	G44R N	L2	2.9
N45L N	loop AB	E46R OE2	L2	3.0
				
K138S NZ	F'	Y43R OH	L2	2.8
S141S O	F'	R96R NH2	L4	2.9
D144S OD1	F'	R96R NH2	L4	2.8
D144S OD2	F'	R96R NH2	L4	3.0
E151S OE1	F'	R191R NH1	L6	2.9
E151S OE2	F'	R191R NH1	L6	2.8
E151S OE1	F'	R191R NH2	L6	2.9
IL-10-IL-10R2 interface				
N18L OD1	A	R191A NH1	L6	2.7
N18L ND2	A	S142A OG1	L6	2.7
N21L ND2	A	N138A O	α A	2.8
R24L NH2	A	N138A OD1	α A	2.7
R24L NH2	A	T133A O	loop Bα A	3.1
D28L OD1	A	K69A NZ	L3	2.8
D28L OD2	A	K69A NZ	L3	3.0
D28L O	A	K69A NZ	L3	3.2
R32L NH1	A	S68A OG	L3	3.3
E81L OE2	loop CD	R46A NH2	L2	3.3
N92L OD1	D	R46A NH1	L2	2.8
IL-10R1-IL-10R2 interface				
E145R OE1	C	N138A OD1	L5	3.2
E145R OE2	C	N141A ND2	L5	2.7
E147R OE2	C	K126A NZ	loop Bα A	3.2
H161R NE2	C'	A124A O	loop Bα A	2.9
K162R NZ	C'	Q110A OE1	A	2.6
K165R NZ	loop C'E	N141A OD1	L5	2.7
H166R NE2	loop C'E	Y166A OH	loop C'E	3.0
S170R OG	E	R121A NH1	B	3.1
S170R O	E	R121A NH1	B	2.6
K185R NZ	F	E132A OE2	loop Bα A	3.2

(*)- SSE: Secondary structure elements. (**)- In INF-γ and IL-10 structures helix F' is a topological equivalent of helix D of other helical cytokines. (***)- Interface areas were calculated with program Surface (CCP4) using spherical probe of the radius 1.40 Å^2^.
